# Promoter-sharing by different genes in human genome – *CPNE1 *and *RBM12 *gene pair as an example

**DOI:** 10.1186/1471-2164-9-456

**Published:** 2008-10-03

**Authors:** Wanling Yang, Ping Ng, Minghui Zhao, Thomas KF Wong, Siu-Ming Yiu, Yu Lung Lau

**Affiliations:** 1 Department of Paediatrics & Adolescent Medicine, LKS Faculty of Medicine, the University of Hong Kong, Hong Kong, PR China; 2 Department of Computer Science, the University of Hong Kong, Hong Kong, PR China

## Abstract

**Background:**

Regulation of gene expression plays important role in cellular functions. Co-regulation of different genes may indicate functional connection or even physical interaction between gene products. Thus analysis on genomic structures that may affect gene expression regulation could shed light on the functions of genes.

**Results:**

In a whole genome analysis of alternative splicing events, we found that two distinct genes, *copine I *(*CPNE1*) and *RNA binding motif protein 12 *(*RBM12*), share the most 5' exons and therefore the promoter region in human. Further analysis identified many gene pairs in human genome that share the same promoters and 5' exons but have totally different coding sequences. Analysis of genomic and expressed sequences, either cDNAs or expressed sequence tags (ESTs) for *CPNE1 *and *RBM12*, confirmed the conservation of this phenomenon during evolutionary courses. The co-expression of the two genes initiated from the same promoter is confirmed by Reverse Transcription-Polymerase Chain Reaction (RT-PCR) in different tissues in both human and mouse. High degrees of sequence conservation among multiple species in the 5'UTR region common to *CPNE1 *and *RBM12 *were also identified.

**Conclusion:**

Promoter and 5'UTR sharing between *CPNE1 *and *RBM12 *is observed in human, mouse and zebrafish. Conservation of this genomic structure in evolutionary courses indicates potential functional interaction between the two genes. More than 20 other gene pairs in human genome were found to have the similar genomic structure in a genome-wide analysis, and it may represent a unique pattern of genomic arrangement that may affect expression regulation of the corresponding genes.

## Background

Genes belonging to the same functional group tend to have similar expression patterns and share expression regulation mechanisms. This was found first in prokaryotes, in which genes of the same functional groups are transcribed into one polycistronic mRNA through an operon structure [1]. It was also found to be true in eukaryotes that genes of similar function tend to be co-regulated and co-expressed. Therefore, gene expression analysis can successfully group genes of the same functional pathways and predict functions for novel genes [2-7]. Genomic arrangement in our genome may affect the expression regulation of different genes, thus understanding of the genomic structures may help us better understand gene expression regulation and gene function.

*CPNE1 *(NCBI GeneID: 8904) is located in human chromosome 20 (20q11.21), and has several alternative splicing forms coding for the same protein of 537 amino acids. *CPNE1 *is expressed in a wide range of organisms, from plants to human. CPNE1 was first identified as a calcium-dependent, phospholipids-binding protein, and it was thought to be involved in membrane trafficking [8]. It contains two calcium-binding, protein kinase C conserved region 2 domains (C2 domains) in the N-terminus and a domain similar to the von Willebrand factor type A domain (A domain) that mediates interactions between integrins and extracellular ligands in the C-terminus. CPNE1 binds phospholipids membranes through the action of its C2 domains that are activated by calcium. Its A domain was shown to bind to a number of intracellular target proteins [8]. While the exact function of CPNE1 is still not clear, it was shown that interaction with CPNE1 may result in recruitment of target proteins to membrane surfaces and regulation of the enzymatic activities of target proteins [9].

*RBM12 *(NCBI GeneID: 10137) contains three exons, with its coding sequence located solely in the large exon 3 of the gene. It codes for a protein of 932 amino acids. Partial *RBM12 *cDNA was cloned first from a brain cDNA library [10], and then from a human colon carcinoma cell line [11]. Abundant mRNA expression of *RBM12 *was shown in all human cell lines studied [11]. The RBM12 protein contains five distinct RNA binding motifs (RBM), two proline-rich regions and several putative transmembrane domains [11]. The RBM domain is an evolutionarily conserved domain that often co-occurs with proline-rich regions. The functions of RBM containing proteins are not known. Some RBM-containing members were found to be involved in apoptosis [12,13]. However, these proteins bear little sequence similarities to RBM12 except that they are all predicted to contain motifs with RNA binding property, and are probably a group of proteins with a broad range of functions.

In a genome-wide analysis of alternative splicing gene variants by alignment of ESTs and human genomic sequences[14], we have discovered that the human *CPNE1 *and *RBM12 *gene often share 5'UTR sequences but do not show any protein coding sequence similarity. Further genomic analysis revealed more than 20 gene pairs with the similar arrangement in human genome. Promoter-sharing between different genes may represent a unique genomic arrangement that regulates co-expression of functionally related genes. In this study, using *CPNE1/RBM12 *gene pair as an example, we showed the conservation of the phenomenon in different species during evolutionary courses. The promoter-sharing and conservation of the 5' UTR sequences of these two genes among multiple species indicate that the two gene products may have some functional connection.

## Result

### 1. Promoter-sharing by different genes in human genome and conservation of the genomic structure for *CPNE1/RBM12 *gene pair during evolutionary courses

From a whole genome analysis for alternative splicing events based on human cDNAs and ESTs [14], we discovered that *CPNE1 *and *RBM12 *share 5'UTR exons and presumably the promoter. Analysis of gene pairs that have transcription initiation sites (TIS) locating in close proximity of each other in the same strand in human genome revealed that many other gene pairs may have similar genomic arrangement (Table 1). Members in these gene pairs usually bear little coding sequence similarity to each other. They are different from the promoter-sharing between adjacent genes locating on the opposite strands through bi-directional promoters. For some of the gene pairs, one gene is a fusion gene of the other gene with an adjacent gene immediately downstream, a genomic arrangement described before [15].

**Table 1:** Gene pairs in human genome that share most 5' exons and promoters with little coding similarities

**Gene 1**	**Chr.**	**Strand**	Gene 1 start *	**Gene 2**	**Gene 2 start**	**Coexpression Correlation Coefficient**	*P*-value (**)	**Note**
LEPOT	1	+	65658906	LEPR	65658905	-0.15	0.73 (103)	

KLHL23	2	+	170259246	PHOSPHO2	170259220	N/A	N/A (0)	

NAT6	3	-	50308836	HYAL3	50305265	0.33	0.16 (103)	

TMED7	5	-	114977101	TICAM2	114942246	0.56	0.04 (105)	

ITGA1	5	+	52119892	PELO	52119530	0.42	0.09 (95)	

MUTED	6	-	7959212	TXNDC5	7826748	0.18	0.30 (107)	

HIST1H2AD	6	-	26306990	HIST1H3D	26304990	0.77	<0.01 (94)	Little coding similarity

LOC552891	9	+	113433486	DNAJC25	113433483	N/A	N/A (0)	One is a fusion gene of two adjacent genes

TRIM6-TRIM34	11	+	5574461	TRIM6	5573922	N/A	N/A	One is a fusion gene of two adjacent genes

HOXC4	12	+	52696908	HOXC6	52696908	0.54	0.04 (103)	some coding similarity; HOXC5 also shares exon 1 with these two genes

ANG	14	+	20222608	RNASE4	20222211	0.77	<0.01 (104)	

SPESP1	15	+	67009918	NOX5	67009917	0.17	0.32 (68)	

SULT1A4	16	+	29373901	GIYD2	29373375	N/A	N/A (0)	

SULT1A3	16	+	30113243	GIYD1	30112717	N/A	N/A (0)	Duplication of the SULT1A4/GIYD2 pair

TNFSF12-TNFSF13	17	+	7393139	TNFSF12	7393098	N/A	N/A (0)	One is a fusion gene of two adjacent genes; structure conserved in mouse

NME1	17	+	46585918	NME1-NME2	46585918	N/A	N/A (0)	One is a fusion gene of two adjacent genes

NUP62	19	-	55101893	IL4I1	55084722	-0.11	0.68 (106)	

PEG3	19	-	62015614	ZIM2	61977731	0.34	0.14 (75)	

ADAT3	19	+	1856416	SCAMP4	1856372	N/A	N/A (0)	

FASTKD5	20	-	3075164	UBOX5	3036218	-0.05	0.61 (105)	Structure conserved in mouse

RBM12	20	-	33700290	CPNE1	33677379	0.47	0.06 (106)	Structure conserved in mouse

BAGE	21	-	10079666	BAGE4	10042712	N/A	N/A	

C22orf29	22	-	18213668	GNB1L	18155937	0.06	0.45 (104)	

ZBED1	X	-	2414454	DHRSX	2147546	0.42	0.09 (103)	

* chromosomal positions are based on NCBI human genome Build 36.3.

** refers to the number of experiments where both genes in that gene pair appeared together.

Expression correlation for the gene pairs was analyzed by data from microarray experiments obtained from Stanford Microarray Database (see method section). For the 24 gene pairs, we have data for 15 pairs, where expression data is available for both genes. Out of these gene pairs, two pairs (ANG and RNASE4; HIST1H2AD and HIST1H3D) showed high expression correlation (r^2 ^= 0.77, *P *< 0.01, respectively). In addition, 7 other gene pairs had an expression correlation coefficient higher than one standard deviation from the mean (Table 1). Six other gene pairs had expression correlation coefficient not different from the mean. We can not determine whether this is due to the quality of the data or that these pairs had poor expression correlation in the involved experiments. Considering that certain issues exist for microarray data, it is safe to conclude that the genomic arrangement does have effect on the co-regulation of the genes for some gene pairs. The result also points out, however, that probably other factors are also playing important roles in the expression regulation of genes in the gene pairs. These may include recognition of the polyadenylation signal of the "shorter" gene in each pair or run-through of the transcription machinery toward the "longer" gene.

**Figure 1:**
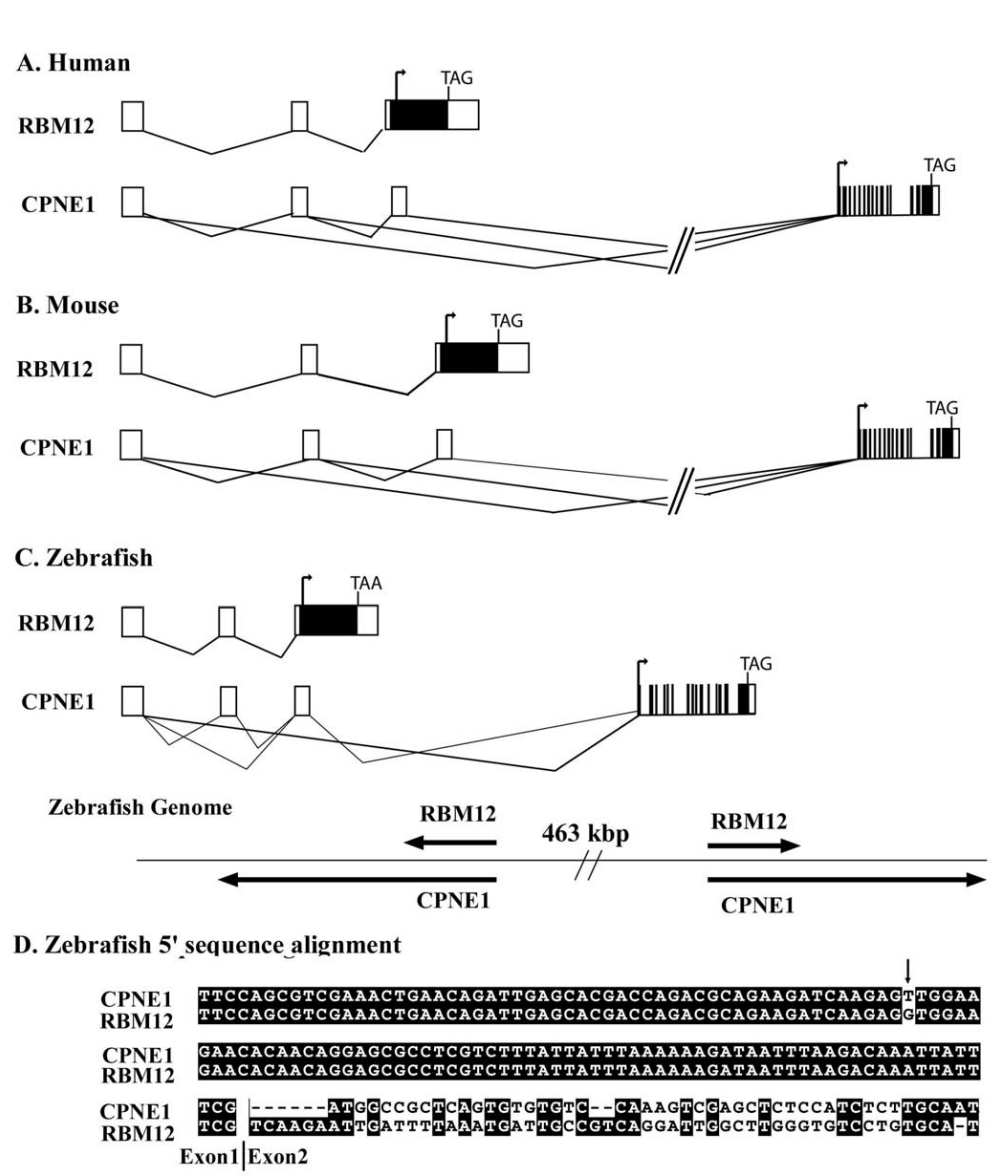
**Gene structure of *RBM12 *and *CPNE1 *and sharing of promoter and non-coding exons between the two genes in human, mouse and zebrafish**. A. Human. Representative cDNAs are [GenBank: NM_152927] for *CPNE1*, and [GenBank: NM_006047] for *RBM12*; B. Mouse. Representative cDNAs are [GenBank: NM_170588] for *CPNE1*, and [GenBank: NM_029397] for *RBM12*; C. zebrafish. There are two copies for each gene in the zebrafish genome, arranged in a head-to-head direction. The duplicated copies of the genes in zebrafish may pose problem to knock-out experiments in the species. D. partial sequence alignment between zebrafish *RBM12 *[GenBank: EB783076] and zebrafish *CPNE1 *[GenBank: NM_199699], labelled as "copine III, like" in NCBI) for the non-coding exon 1 and succeeding sequences. The one nucleotide difference (indicated by arrow) could be due to either polymorphism on the site or a sequencing error. Other sequences that share first exon with [GenBank: EB783076], and therefore may also share promoters with *CPNE1 *cDNA [GenBank: NM_199699] include: [GenBank: EB832449, DT271069, DT222776 and EB775439] etc.

We used *CPNE1 *and *RBM12 *gene pair as an example to further study this genomic arrangement using bioinformatics tools. The promoter-sharing between the two genes in human and mouse is obvious from gene annotations from both NCBI (NCBI human genome Build 36.3, see Additional file 1, Figure 1) and Ensembl (data not shown), but whether this arrangement is conserved in other species is not clear. Sharing of the most 5' exons between these two genes is a common pattern revealed by many of the transcripts of these two genes (both ESTs and cDNA), indicating that the sharing is a common phenomenon rather than a rare transcription event. A combination of various methods was used to analyze the orthologous genes in various species. These include searching the NCBI_nr database, Swiss-Prot, and dbEST, as well as searching the genomic sequences of model species  to identify the genomic sequences of the orthologous genes for *CPNE1 *and *RBM12*. The sharing of 5' UTR exons and the promoter region between the two genes was confirmed in mouse, rat, chimpanzee, rhesus monkey, and zebrafish. Distinct full-length cDNA sequences were used to align with the respective species genomes to determine the gene structures (Figure 1). Two zebrafish cDNA/EST sequences respectively representing *CPNE1 *and *RBM12 *were aligned, demonstrating the sharing of the first exon as well as the divergence afterwards between the two genes (Figure 1D). Although [GenBank: EB783076] is an unannotated EST sequence, there are other zebrafish ESTs for *RBM12 *from different tissues and sources supporting the sharing of the first exon with annotated zebrafish *CPNE1 *cDNA [GenBank:NM_199699], such as [GenBank:DT222776, EB775439, EB832449, and DT151375] (see Additional Figure 2). We have attempted but found no evidence that the orthologous genes for *CPNE1 *and *RBM12 *in more primitive species, such as *C. intestinalis*, *C. elegans*, or yeast would share the same genomic locus and promoter region.

### 2. Expansion of the two gene families during evolutionary courses and its relationship to the promoter-sharing

**Figure 2:**
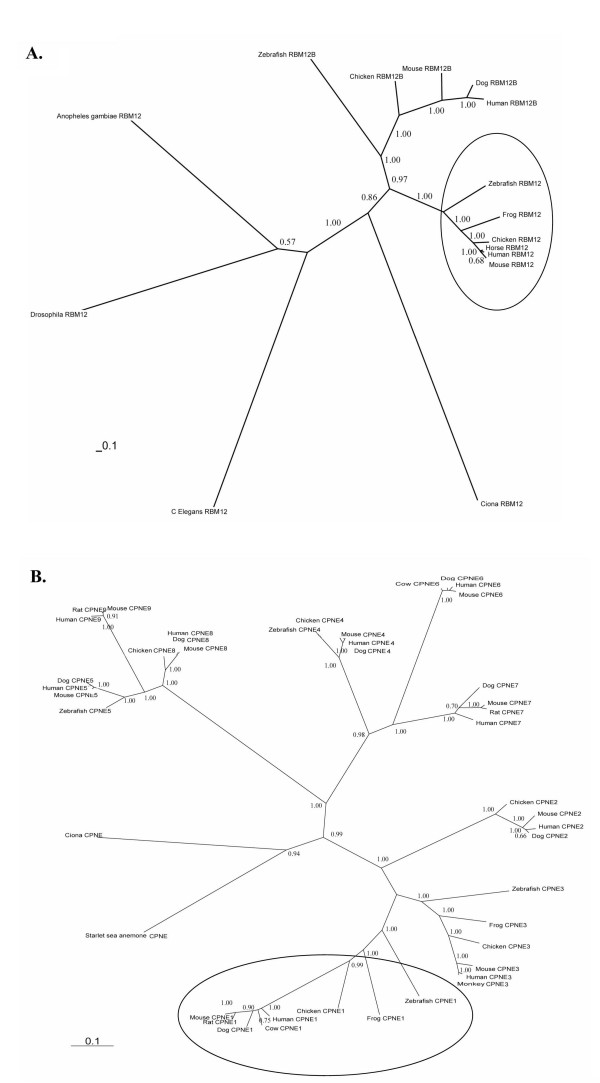
**Phylogenetics analysis of expansion of the CPNE and RBM12 families**. Protein sequences of the two gene families from various species were aligned using ClustalX. The aligned sequences were analyzed by MrBayes 3.1.2 for their phylogenetic distances and displayed by TreeView. The numbers shown on each branch are the posterior probabilities of the phylogenetic relationship. The circle marked the genes that shared promoters between CPNE1 and RBM12.

In an effort to examine the evolutionary changes of the two gene families, we have extracted and compared the predicted protein sequences of the paralogs and orthologs for these two genes from various species. Protein sequences for these two genes in different species were predicted from corresponding cDNA or EST sequences. The sequences were aligned by the multiple sequence alignment program ClustalX, and the alignment file was used for predicting the phylogenetic distances of different proteins using MrBayes (Figure 2). It is clear from the phylogenetic tree that, during the evolutionary courses starting from fish, *RBM12 *family expanded to *RBM12 *and *RBM12B*, and *CPNE *family expanded to 9 paralogs from *Copine I *to *IX*. Sequences from other species, such as *C. elegans *and *C. intestinalis *are much more divergent and do not group with any of the subgroups in either of the two gene families. It seems that the expansion of the two gene families started with fish, and *CPNE1 *and *RBM12 *may evolve together functionally with conserved promoter-sharing and co-regulation. From the phylogenetic distances, it is also interesting to note that among the paralogous *CPNE *genes, mammal *CPNE1 *sequences diverged very much from their counterparts in chicken, frog, and zebrafish (circled group in Fig. 2B), more so than in other *CPNE *genes, indicating that mammal *CPNE1 *may have evolved new functions much different than those in other species.

From the sequence alignment of all the homologous proteins, we noticed that there is limited sequence similarity between human RBM12 and RBM12B, except for the two terminus regions and the N-terminus region in particular, for which they are almost identical (see Additional Figure 3A). This is consistent with the conservation of the N-terminus region among RBM12 orthologs from different species ranging from zebrafish to human, with near complete conservation for the first 90 N-terminal amino acids and diverged afterwards in fish and frog (see Additional Figure 3B). This region does not coincide with the RBM domain or match with any other conserved domains in the protein databases. In contrast, the relatively higher conservation among the paralogs and orthologs of human CPNE1 is across the full length of the protein, with no particular regions standing out (data not shown). The conserved regions between RBM12 and RBM12B could be the regions involved in conserved functions between the two genes, while diverged sequences may indicate evolvement of new functions after gene expansion.

### 3. Detection of co-expression of the two genes from the same promoter in human and mouse

**Figure 3:**
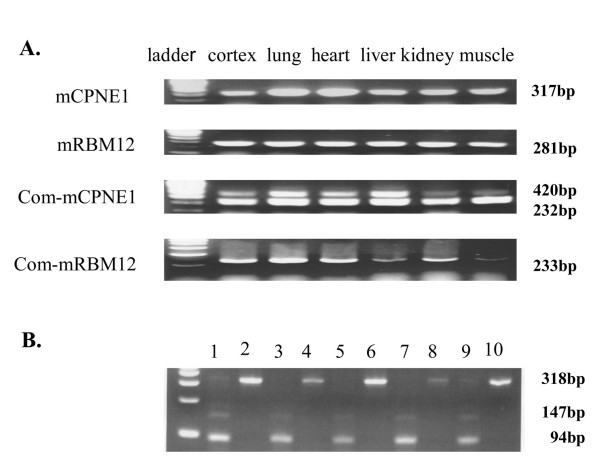
**Reverse-Transcription PCR for the co-expression of *RBM12 *and *CPNE1 *in mouse tissues and human PBMC**. A. Expression of the two genes in various mouse tissues. Top panel (mCPNE1): *CPNE1 *expression detected by *CPNE1*-specific primer pair; Second panel (mRBM12): PCR results from RBM12-specific primers; third panel(Com-mCPNE1): amplification from common forward primer from the non-coding exon2 and *CPNE1*-specific reverse primer from exon 4, the two bands reflect alternative splicing forms including/excluding exon 3; Lower panel(Com-mRBM12): PCR from common forward primer from non-coding exon2 and RBM12-specific primer from the *RBM12*-specific region of exon 3. **B**. Expression of the two genes in Human PBMC. Lane 1, 3, 5, 7, 9 are expression of *CPNE1 *from five individual blood donors, as amplified by a common forward primer in exon1 and *CPNE1*-specific reverse primer in exon 4. Lane 2, 4, 6, 8, and 10 are expression of *RBM12 *from the five individuals as amplified by common forward primer from exon 1 and a RBM12-specific primer in exon 3. The different bands in *CPNE1 *amplification reflect alternative splicing forms.

In order to experimentally examine the expression profile of the two genes and the sharing of promoter and non-coding exons, we have examined the expression of the two genes in human peripheral blood mononuclear cells (PBMC) from five individuals and in multiple mouse tissues using RT-PCR (Figure 3). The results verified the expression of the two genes from a common promoter in both species, although the experiment does not prove their co-expression from the same cells. The result is also consistent with reports on the ubiquitous expression of these two genes[8,11,16]. Expression of the two genes was verified by sequencing some of the PCR products. The expression levels of the two genes were not compared by any quantitative measure, but EST analysis indicates similar expression levels between the two genes (data not shown).

### 4. Alternative splicing and sequence conservation of 5' UTR region in multiple species

**Figure 4:**
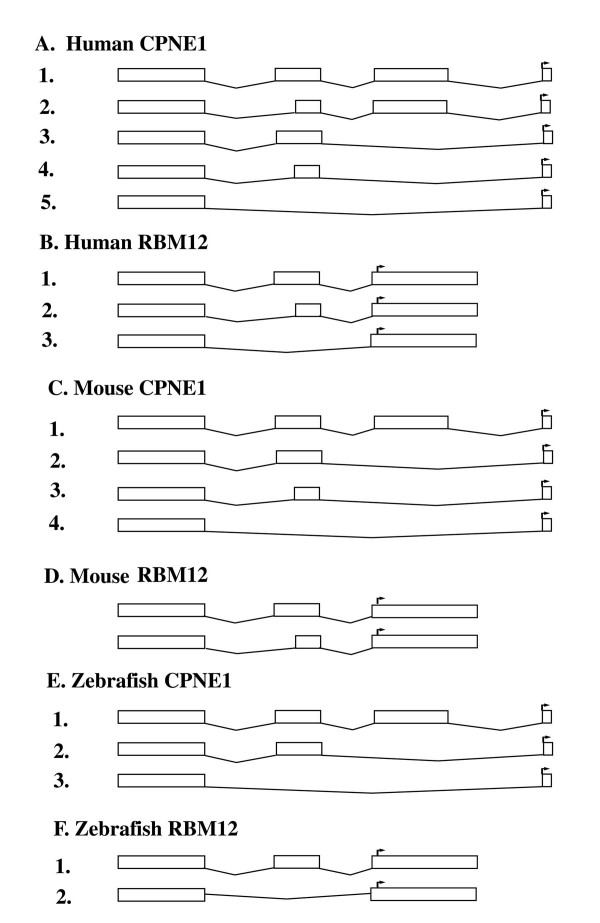
**Alternative splicing forms of *CPNE1 *and *RBM12 *in human, mouse and zebrafish in the 5'UTR**. GenBank accession nos. for representative cDNA or EST sequences for Human *CPNE1*: 1. [GenBank:NM_152930], 2. [GenBank:NM_152931], 3. [GenBank:NM_152927], 4. [GenBank:NM_152928], 5. [GenBank:NM_152925]; Human *RBM12*: 1. [GenBank:NM_006047], 2. [GenBank:NM_152838], 3. [GenBank:AB018308]; mouse *CPNE1*: 1. [GenBank:CF742269], 2. [GenBank:CN693202], 3. [GenBank:NM_170588], 4. [GenBank:NM_170590]; mouse *RBM12*: 1. [GenBank:BC052473], 2. [GenBank:AF393216]; zebrafish *CPNE1*: 1. [GenBank:XM_001338967], 2. [GenBank:EB992764], 3. [GenBank:XM_696989]; zebrafish *RBM12*: 1. [GenBank:DT275702], 2. [GenBank:EB783076].

In addition to the co-regulation of the expression of these two genes through shared promoter region, the two genes also share non-coding exons, which are also conserved during evolutionary courses. We have examined the alternative splicing patterns of the two genes in different species, especially focusing on the 5'UTR where most alternative splicing forms are derived. As shown in Figure 4, most of the alternative splicing forms and the gene structure in the 5' UTR are well conserved between human, mouse and zebrafish, indicating that the sequences in the 5' UTR may have a functional role.

**Figure 5:**
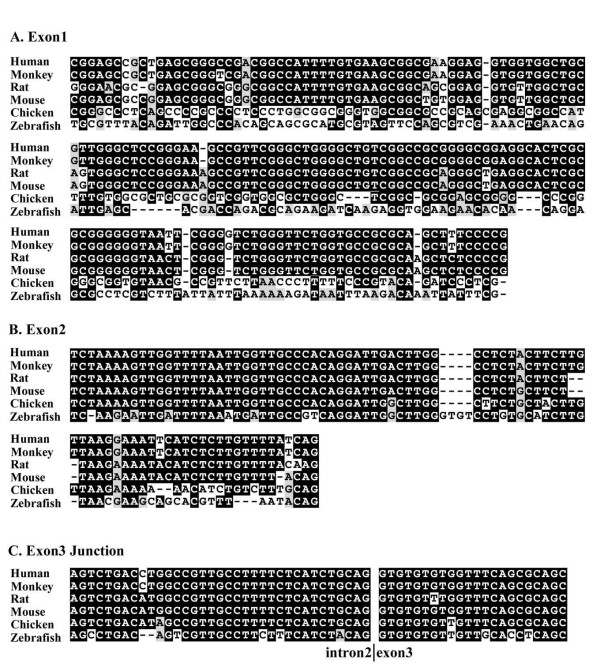
**Sequence alignment of conserved regions upstream of the coding sequence of *CPNE1 *and *RBM12 *among multiple species**. **A**. conservation of exon 1; **B**. conservation of exon 2; **C**. conservation in both the splicing acceptor region of intron2 and the non-coding sequence of exon 3.

Sequence conservation among different species, especially species that are set apart by hundreds of million years of evolution, may indicate strong selection constraint and probably functional implications. Next we compared the promoter region and 5'UTR sequences from multiple species and tried to identify the motifs that remain conserved during evolutionary courses. Interestingly, sequences from the three non-coding exons for these two genes showed strong sequence conservation among different species. The only other region that showed high level conservation is the splicing acceptor of intron2 (Figure 5), with a conservation level probably higher than most splicing acceptor regions, indicating a possible role in alternative splicing regulation.

Secondary structures in the 5'UTR are known to regulate translation efficiency, and long 5'UTR has been reported to associate with low translation efficiency [17,18]. Analysis for secondary structure formation predicted from mammal *RBM12 *5'UTR sequences showed a possibility of stable secondary structures of the 5'UTR region (see Additional file 1, Figure 4). The conserved sequences, as well as the potential secondary structure formation may play a role in expression regulation, alternative splicing, and translation efficiency.

Comparative sequence analysis of the immediate upstream region of the gene pair (1,000 bp from TIS) in different species did not reveal strong sequence conservation as observed in the 5'UTR region, except for the immediate upstream sequences (-1 to -300 bp) between mouse and rat (see Additional file 1, Figure 5). However, when combining sequence analysis among different species and transcription factor binding site search using rVISTA [19], we found that many predicted transcription factor binding sites corresponded in the sequence alignment between different species (aligned TF binding site hits, see Additional file 1, Figure 6). So it is possible that although the exact sequence changed among species, transcription factor binding sites may still be conserved. For between mouse and rat, the immediate upstream 300 bp region where the core promoter may reside demonstrated a strong sequence conservation as well as transcription factor binding site correlation (conserved TF binding site hits, see Additional file 1, Figure 6).

## Discussion

### 1. Coexpression of genes and its functional implications

In eukaryotes, genes that belong to the same functional groups or whose products physically interact are more likely to share similar expression patterns and regulation [2-7]. The promoter-sharing between *CPNE1 *and *RBM12 *and the conservation of this phenomenon during evolutionary courses probably reflect a selection constraint to keep the two genes co-regulated, which in turn suggest of a functional relationship between these two genes. It is possible that the potential interaction of CPNE1 and RBM12 reflects a new function evolved starting from fish and maintained in mammals.

Different genomic arrangements in eukaryotes exist to ensure co-regulation of different genes and their co-expression. In the setting of bidirectional promoters, two genes are arranged in a head-to-head pattern with their TIS close to each other (within 1 kb) in the same genomic locus. This arrangement provides a mechanism of co-regulation of two different genes [20], although the promoter may have different activity toward regulating the genes on the opposing strands. Two human genes, *HADHA *and *HADHB*, which encode the subunits of an enzyme complex (trifunctional protein) involved in mitochondrial beta-oxidation of fatty acids, are controlled by a bidirectional promoter. The 5' flanking region common to the two genes was shown to have bidirectional promoter activity and controls the expression of both genes [21]. It was also shown that many cancer genes are regulated by bidirectional promoters [20].

Many paralogous genes are derived from genomic duplication. They are usually involved in the same functional activities. Some of these genes may share a common promoter that ensures their co-expression and co-regulation. It was reported that a common promoter controls the transcription of a pre-mRNA comprising exon sequences of two transcription factor genes, *hoxb3a *and *hoxb4a *in zebrafish. It was suggested that the unique gene structure is to provide a novel mechanism to ensure overlapping, tissue-specific expression of both genes in the posterior hindbrain and spinal cord [22]. Rnf33 and Rnf35 are two RING finger protein genes that are transcribed temporally in the preimplantation mouse embryo, predominantly at the two-cell embryonic stage. The two genes are apparently transcribed from the same putative promoter, presumably ensuring their co-expression in a spatial and temporal manner [23]. Another arrangement that may involve co-regulation of different genes is nested genes, in which a gene usually resides in an intron of a host gene [24,25]. However, this arrangement is more likely to result in interference in the expression of the genes [25], rather than coordinated expression.

Of course, for majority of the co-regulated genes in eukaryotes, they could reside on different chromosomal regions and are probably regulated by binding of common transcription factors or feedback processes. It was shown that genes with similar functional annotations are more likely to be bound by a common transcription factor [6]. It was reported that most of the genes in the oxidative phosphorylation system co-express in both human and mouse, and subunits of each complex tend to have tighter co-expression within the same complex than with subunits of other complexes in the system. Common promoter elements and transcription factor binding sites are proposed to be factors in the co-regulation of these genes [26].

Reversely, it has been proposed that highly coordinated expression of genes is likely to indicate functional relationship or even physical interaction of the gene products [27]. It has been found that in the budding yeast, clustering gene expression data efficiently groups together genes of known functional groups [2]. It was shown that co-regulated genes have a strong tendency to belong to the same protein complex in prokaryotes, and was shown also to be true in yeast and *C. elegans *[3]. Co-expression relationship has been used to assign functional predictions to uncharacterized genes and has identified potential new members of many existing functional categories [4]. In a similar study, it has been shown that quantitative transcriptional co-expression is a powerful predictor of gene function based on data from microarrays in 55 mouse tissues [5]. It was reported that for at least 75% of the conserved co-regulated gene pairs, physical interactions between the encoded proteins have been demonstrated [28]. These proteins include ribosomal proteins, RNA polymerase subunits, ATP synthase subunits, transporter subunits, various enzyme-subunits, and cell-division proteins. Teichmann *et al. *[3] concluded that genes for which co-regulation is conserved across distantly related genomes are very largely, if not entirely, those that physically interact to form stable complexes in both prokaryotes and eukaryotes.

Niehrs and colleagues [29] raised the theory of co-evolution of function and expression, or co-evolution of promoter and coding sequences. Apart from energetic economy, interacting gene products frequently need to assemble stoichiometrically or may require co-translation for forming a complex, which is promoted by co-expression. Therefore, components of supramolecular complexes will probably be organized in synexpression groups. Snel and colleagues[30] showed that in the case of gene duplication after speciation, one of the two inparalogous genes tends to retain its original co-regulatory relationship, while the other loses this link and is presumably free for differentiation or sub-functionalization.

Although it could be argued that sharing of 5'UTRs may not necessarily provide evidence of promoter sharing, aligning of cDNAs and ESTs of the two genes showed that in majority of the cases, they have a common exon 1 with the identified most 5' sequences in close proximity of each other, which is a strong indication that they probably share the same promoter with the same or close TIS (see Additional Figure 7). It is possible that alternative promoters may also be used in addition to the shared common promoter. For human *CPNE1*, [GenBank:NM_003915] represents a transcript with an alternative exon 1 but with the same coding sequences; there is no evidence that human *RBM12 *uses an alternative promoter. The cDNA and EST sequences seem to support that the predicted common promoter is the major promoter in both human and mouse, which may not be the case in zebrafish, as the evidence of promoter sharing only came from a few EST supports (see Additional file 1, Figure 2). As more data on the expression of the two genes become available, it could be determined whether or not the shared promoter between the two genes is the major promoter in fish and in other non-mammal species.

### 2. Sequence conservation in the 5' UTR region

Comparison of the non-coding sequences common to *CPNE1 *and *RBM12 *revealed high level of sequence identity among species ranging from fish to human comparable to that of the coding regions. The conservation of both gene structure (Figure 4) and 5' UTR sequences (Figure 5) may indicate a role in expression regulation, alternative splicing, or translation regulation.

It has been reported that about 70% of the sequences conserved among multiple species resides within non-coding regions with no known function [31], and much of these non-coding conservation reside in the UTRs. The 5' UTR sequences may affect translation efficiency [17,18]. The efficiency of translation initiation is largely governed by the composition and structure of the 5' UTR of the mRNA, which is determined by both its length and its sequence [32]. Stable secondary structure and small upstream open reading frames within a 5' UTR can profoundly inhibit protein translation. Most highly expressed mRNAs have relatively short (20–100 nucleotides) 5' UTRs that lack upstream ORFs and extensive secondary structures [33]. In contrast, mRNAs encoding growth factors, transcription factors, oncoproteins and other regulatory proteins have been found to be poorly translated and often have long, highly structured 5' UTRs with multiple upstream ATGs [17,18]. 5' UTR sequences are also shown to play roles in alternative splicing and expression regulation [34,35]. The long, conserved 5'UTR sequences and the potential of forming a stem loop secondary structure in this locus may indicate a role of this region in the regulation of these two genes. The unusual conservation of splicing donor sequences in intron2 (Figure 5C) may take part in alternative splicing of different forms. It will be interesting to see what role these sequences play in the regulation of the two genes through wet lab experiments.

#### 3. Role of polyadenylation and alternative splicing in the expression of the two genes

An interesting question is at what point the expression of either *CPNE1 *or *RBM12 *mRNA is determined. The decision is probably not lying on the transcription initiation since the two genes apparently share the same promoter region. It is likely that polyadenylation or alternative splicing, or the cooperation of the two processes determines which gene to express. Binding of polyadenylation machinery and termination of transcription may both be involved in the process.

Cleavage/polyadenylation specificity factor (CPSF) plays a central role in pre-mRNA 3' cleavage and poly(A) addition. CPSF appears to travel with RNA polymerase II until reaching the polyadenylation element (AAUAAA), where it may dissociate and define the poly(A) site [36]. A functional mRNA polyadenylation signal was shown to be required for transcription termination by RNA polymerase II [37]. It is suggested that perhaps dissociation of the poly(A) factors influences the ability of Pol II to elongate, thereby providing a partial explanation for the requirement of a functional poly(A) site for transcription termination [38]. There are putative AAUAAA signal both at the end of *RBM12 *exon 3 and the last exon of *CPNE1*, which are 23 kb apart from each other. Although transcription usually continues beyond the poly(A) site in both viral and cellular genes, terminating as much as several kilobases downstream from the poly(A) site [39-41], it is likely that transcription termination is playing some roles in the determination of which gene mRNA to express in this case. It is possible that the recognition of the AAUAAA site at the end of *RBM12 *may facilitate the termination of transcription, and may work together with splicing machinery and destine the transcription into generating *RBM12 *mRNA. Or a suppression of recognition of the *RBM12 *AAUAAA may facilitate the transcription machinery to proceed toward *CPNE1 *exons downstream and lead to the synthesis of *CPNE1 *pre-mRNA. Examinations on whether there are two distinct populations of pre-mRNA corresponding to either of the two genes will help answer this question.

### 4. The functions of *CPNE1 *and *RBM12*

CPNE's biological role is still unclear. It has been postulated that they may be involved in exocytosis [8] and phagocytosis [16]. In green plants, mutation of a *CPNE *gene leads to alterations in plant size, stress responses and apoptosis [42,43]. CPNEs were found to be required for cytokinesis, contractile, vacuole function and development in Dictyostelium[44]. Tomsig and colleagues [9] reported that the A domains of human copines mediate the binding of copines to target proteins. The target proteins detected interacting with CPNE1 by a yeast two-hybrid system include protein phosphatase 5 catalytic subunit, Myc binding protein 2, ubiquitin-conjugating enzyme E2O, Radixin, and beta-actin, with more partners found with the in vitro pull-down assay. The copines are shown to be able to recruit these target proteins to phospholipids surfaces, suggesting that they may regulate their activities and localization in cells in response to changes in intracellular calcium. And a possible function of the copines may be to confer calcium regulation on intracellular signalling pathways such as growth control, exocytosis, mitosis, apoptosis, gene transcription and cytoskeletal organization.

Recent studies also show that CPNE1 could be involved in TNF-α-dependent expression of NF-κB. A copine dominant-negative construct was found to reduce the activation of the transcription factor NF-κB by TNF-α in HEK293 cells [45]. The introduction of calcium into HEK293 cells was found to enhance TNF-α-dependent activation of NF-*κ*B. This effect of calcium was completely blocked by the copine dominant-negative construct. However, Ramsey and colleagues [46] subsequently showed that CPNE1 is a novel repressor to inhibit NF-κB transcription through physically interacting with p65. Despite the controversies on the exact role of CPNE1, it seems certain that CPNE1 is playing an important role in TNF-α-stimulated NF-κB transcription. TNF-α and NF-κB are involved in a wide range of cellular functions, and it would be interesting to find out whether the proposed interaction of CPNE1 and RBM12 play any role in these processes.

Little is known about the function of RBM12 protein. RBM12 was detected as upregulated in Meibomian cell carcinoma, a malignant tumour of themeibomian glands located in the eyelids[47]. RBM3 and RBM5 were found to suppress apoptosis [12,48]. Sutherland and colleagues[13] raised the question that maybe all RBM proteins are involved in apoptosis regulation. Both *CPNE1 *and *RBM12 *seem to be ubiquitously expressed [8,11]. RBM12 contains putative transmembrane domains [11], although the cellular localization of the protein was never elucidated. CPNE1 does not contain predicted transmembrane domains, but binds to phospholipids membranes upon calcium activation. So it is likely that the two proteins may interact on the plasma membrane upon calcium activation. The potential interaction of the two gene products may play roles in membrane trafficking, growth control or apoptosis.

### 5. Genomic analysis in hypothesis forming

To our knowledge, except for certain paralogous genes locating in the same locus due to chromosomal fragment duplication, there is no report that different genes would share the same promoter in the same orientation. Our findings may represent a new phenomenon in gene expression regulation. It should be noted that gene pairs listed in Table 1 could be an under-representation of this kind of genomic arrangement, and in-depth cDNA and EST sequencing may reveal more gene pairs sharing promoters.

With the knowledge of complete human genome and the rapid pace of cDNA sequencing, many new genes have been discovered. However, elucidating the functions of these genes has proven to be difficult and in a much slower pace. The availability of genomic sequences of model species and high-throughput expression data makes it possible to use genomic analysis in predicting gene functions and guiding experimental designs in elucidating gene functions. Our findings are somewhat unique in that the two genes show no sequence similarity, yet maintain a strong conservation in expression regulation elements. This information points to a probable scenario that the two genes may functionally associate, or even physically interact. These are two genes with undefined functions but all the evidence is pointing to important roles in a wide range of cellular activities. It will be interesting to see wet lab experiment results testing this hypothesis and we expect more genomic analysis-guided researches in the effort to understand gene expression regulation and functions of novel genes.

A note of caution is that sharing of promoter may not necessarily mean co-expression of the gene pairs. As discussed above, polyadenylation regulation and/or splicing machinery may still determine differential expression of the genes. The effect of this genomic arrangement on gene expression regulation, and on the functional implications for the gene pairs listed in Table 1 warrant further investigation through gene expression analysis and functional characterizations.

## Methods

### Identification of promoter-sharing in human genome

To identify gene pairs sharing the first exon in human genome, we first located the genes in the same strand and whose genomic regions overlap. Information on those genes, including the starting/ending position, chromosome and strand was downloaded from NCBI website (Human genome Build 36.3). About 200 pairs of genes were identified as locating in the same strand, and their gene regions on the chromosome overlap with each other. Gene pairs with starting positions differ by less than 1000 bp were kept for further analysis. Around 50 gene pairs are selected for further checking after this stage. Each of the remaining gene pair was further examined using their corresponding mRNAs and ESTs and MapViewer annotations. 24 gene pairs that share the same first exon and have different coding sequences were selected (Table 1).

### Analysis of co-expression of the gene pairs by microarray data

Several thousand sets of human gene expression data were downloaded from Stanford Microarray Database [49]. Data was retrieved in the format of log (base 2) values of R/G normalized ratio. For most of the gene pairs listed in Table 1, experimental data was available where both genes appeared at the same time. For these pairs, we have calculated the coexpression correlation coefficient between the genes in each pair.

In order to better understand the statistical significance of the expression correlation, we have randomly chosen 100 sets of microarray experiment data from the database and selected 150 genes to calculate the distribution of correlation coefficient between random gene pairs. We calculated the average (μ) and the standard deviation (σ) of correlation values of all _150_C_2 _= 11175 possible pairs among these sets of data. Assuming the correlation coefficients are distributed normally, the *P *value of a correlation coefficient (v) was then calculated as:

P(z > (v-μ)/σ)

The mean correlation coefficient between the randomly chosen pairs is 0.029 with a standard deviation of 0.293. *P *value will be <= 0.01 when the correlation coefficient is > 0.7; and the *P *value is <= 0.05 when the correlation coefficient is > 0.5. Based on the nature of the data, only the probability of positive correlation between the genes in each pair is considered. Considering the possibility of real expression correlation among the random pairs formed by the 150 genes chosen, this statistical threshold is likely to be conservative.

### Identification of human *CPNE1 *and *RBM12 *orthologous and paralogous genes

A combination of different methods was used to collect cDNA and protein sequences for orthologous and paralogous genes of human CPNE1 and RBM12. Some already annotated members were collected from NCBI Entrez Gene  and Swiss-Prot . Otherwise, human CPNE1 and RBM12 protein sequences were used as templates to search for orthologous genes using tblastn program[50] from different genomes of model animals, such as *C. intestinalis*, *C. elegans*, drosophila, zebrafish, mouse, rat, and rhesus monkey, etc. The aligned genomic sequences from these genomes showing highest alignment qualities (high percentage of sequence similarity over a significant stretch) were selected and used as templates in further Blast search of expressed EST sequences (dbEST) and cDNA sequences (nr database from NCBI) for best matches of cDNA sequences in different species. Protein sequences were predicted from identified EST or cDNA sequences using "ORF Finder" from NCBI . The protein sequences are used in subsequent sequence alignment by ClustalX and phylogenetic distance analysis by MrBayes. Sometimes a direct similarity search using Blastn was also used to identify the paralogs and orthologs of these two genes by searching NCBI_nr and dbEST databases.

Different representative forms of cDNA sequences were selected to determine the respective gene structures by aligning the most complete cDNA sequences from each form with their respective genomic sequences using Blastn.

### Phylogenetic tree construction

Protein sequence alignment of the homologous proteins was performed using ClustalX [51,52] by Gonnet series protein weight matrix and standard parameters. The alignment data was saved as nexus format and used in the succeeding phylogenetics analysis. MrBayes v3.1.2 [53,54] was used for phylogenetics distance analysis. The analysis was performed according to standard procedures defined by the program until standard deviation of the split frequencies reaches below 0.01. The final phylogenetic distance was displayed using TreeView [55].

### Identification of multi-species conserved sequence

All the syntenic genomic sequences in this locus from different species, from 1 kb upstream of TIS to the coding region of RBM12, were aligned using program DIALIGN-T. DIALIGN-T is a segment-based approach, which uses a greedy optimizations procedure for multiple sequence alignment [56]. Conserved sequences in this genomic region from multiple species were identified and displayed using Boxshade (Kay Hofmann, Michael D. Baron Institute for Animal Health, U.K.).

Sequences in regions conserved among multiple species were used to predict potential secondary structure formation using Alifold program [57]. Consensus secondary structure prediction for the 5' UTR sequence of the two genes (including exon 1, exon2, and non-coding sequence of exon 3) was shown in Figure 7.

### RNA extraction and reverse transcription PCR

Peripheral blood mononuclear cells (PBMCs) (about 10^6 ^cells) from five healthy Red Cross blood donors were used and total RNA was extracted from the cell pellets using Trizol LS Reagent (Invitrogen, San Diego, CA). Total RNA from different tissues of healthy C57BL6/J mice was also extracted using the same method described above. RNA sample quality was determined by visualization of the 18s and 28s RNA bands under UV light after agarose gel electrophoresis. cDNA was generated from the extracted total RNA by reverse-transcription using SuperScript II kit with oligo-dT as primer (Invitrogen, San Diego, CA) according to the manufacturer's instructions. PCR conditions used are 96°C 5 mins, followed by (96°C 30 sec; 58°C 30 sec; 72°C 1 min) for 40 cycles, then followed by 72°C 7 mins.

Human primers used in this experiment:

Common Forward Primer: 5'TAATTCGGGGTCTGGGTTCTGGT3'; reverse primer for CPNE1: 5'ATGAGATGGTCACAGGAAATGGAC3'; reverse primer for RBM12: 5'CATACCAAGCCTTGCATCTTCATC3'; CPNE1-specific primers: forward primer: 5'ATCACGGTCTCAGCTCAGGAATTA3'; reverse primer: 5'ATTGCACCTGGATGGGTGTGCT3'; RBM12-specific primers: forward primer: 5'GCCCTTTACTGTGTCTATTGATGAG 3'; reverse primer: 5'TGGATGCATTAATCACAGCAATATG 3'.

Primers used for mouse tissues:CPNE1-specific primers: forward: 5'TGACCTTACCCTTGATGTTGAAGCCT3'; reverse: 5'ATAGTCTGAGCAGCGCACCTGAATG3'; RBM12-specific primers: forward: 5'-GGTGCAGAACATGCCTTTTACTGTA-3'; reverse: 5'TGGATGCATTAATCACAGCAAAATAA-3'; common forward primer: 5'-GGATTGACTTGGCCTCTGCTTCTTAA-3'; reverse primer-CPNE-specific: 5'-AGAGTCTTGGAGAACTCAGGGCTTGA-3'; reverse primer-RBM12-specific: 5'-CTTGCATCTTCATCAGTGGCAAAAAC-3'.

## Conclusion

*CPNE1 *and *RBM12 *are two genes with unknown functions. Genomic analysis revealed that the two genes share 5'UTR exons and presumably the promoter region. This phenomenon is conserved in mammals and can be traced to zebrafish. Both the sequences of 5'UTR and the gene structure are well conserved during evolutionary courses, indicating that co-regulation of the two genes may have some functional constraint. The two proteins may functionally interact to play a role in calcium-induced signalling. There are many other gene pairs in human genome showing the same genomic arrangement (Table 1), representing one of the genomic structures affecting gene expression regulation.

## Abbreviations

*CPNE1*: *copine I*; *RBM12*: *RNA binding motif protein 12*; TIS: Transcription initiation site; PBMC: Peripheral blood mononuclear cells; CPSF: Cleavage/polyadenylation specificity factor; EST: Expressed sequence tags.

## Authors' contributions

WY conceived of the study, carried out the bioinformatics analysis, and drafted the manuscript. PN and MZ carried out the RT-PCR experiments and participated in manuscript revision. TKFW and SMY carried out the genomic analysis that led to the discovery of the promoter-sharing between CPNE1/RBM12 gene pair and subsequent analysis of other gene pairs with similar arrangement in human genome, as well as the coexpression correlation of these gene pairs. YLL participated in the design of the study and in manuscript revision. All authors read and approved the final manuscript.

## Supplementary Material

Additional file 1 **Additional Figures 1–7.** Click here for file
