# Supplementary material for: Promoter-sharing by different genes in human genome – CPNE1 and RBM12 gene pair as an example
Source: BMC Genomics. 2008 Oct 3;9:456. doi: 10.1186/1471-2164-9-456 (PMC2568002; doi:10.1186/1471-2164-9-456)

Supplementary Figure 1. Mapviewer display of CPNE1 and RBM12 in human and mouse genome

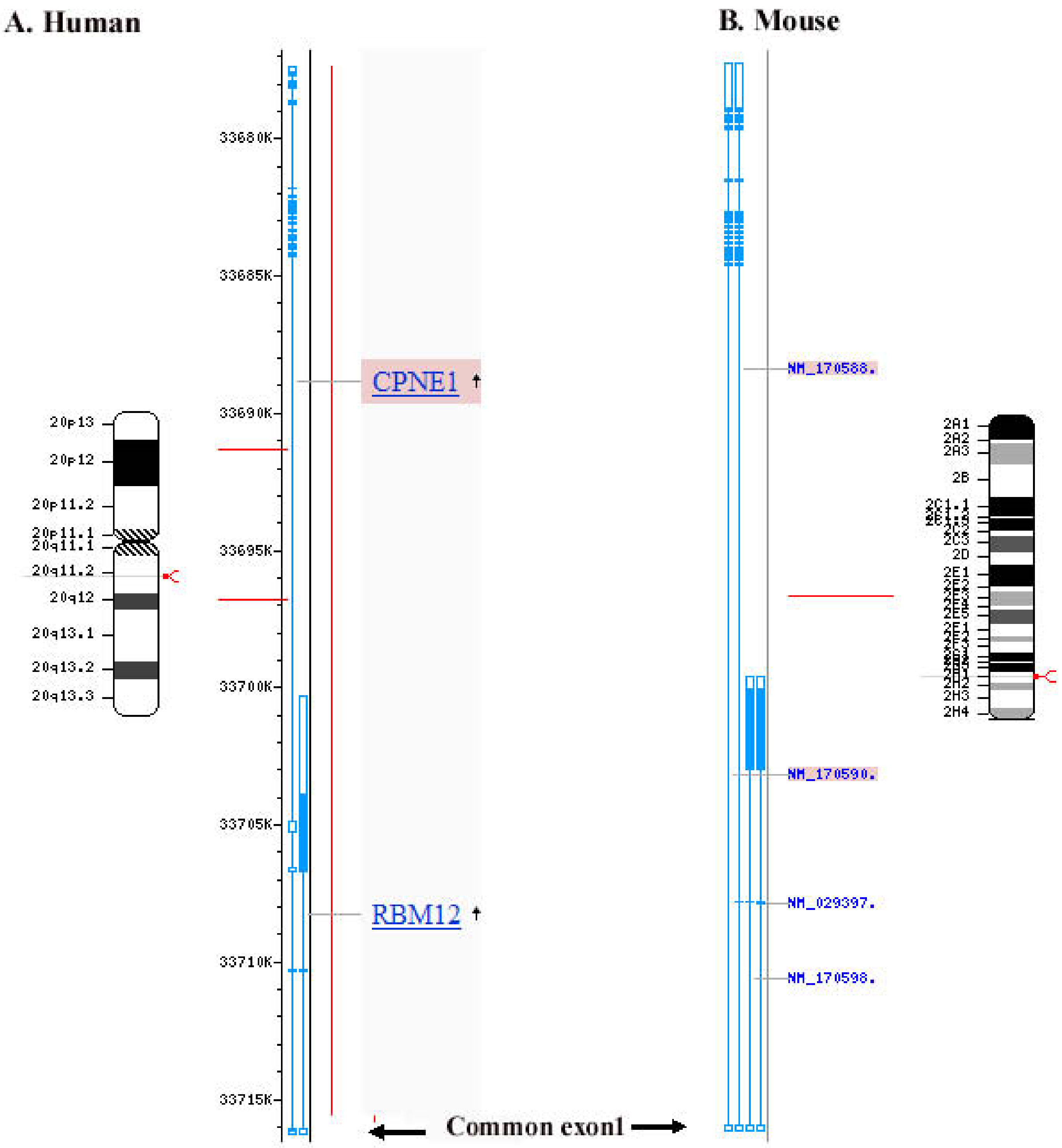

Supplementary Figure 2.

Zebrafish RBM12 EST sequences aligning with Zebrafish CPNE1 cDNA, NM_199699

--Evidence of sharing most 5' exon and therefore promoter between the two genes in zebrafish

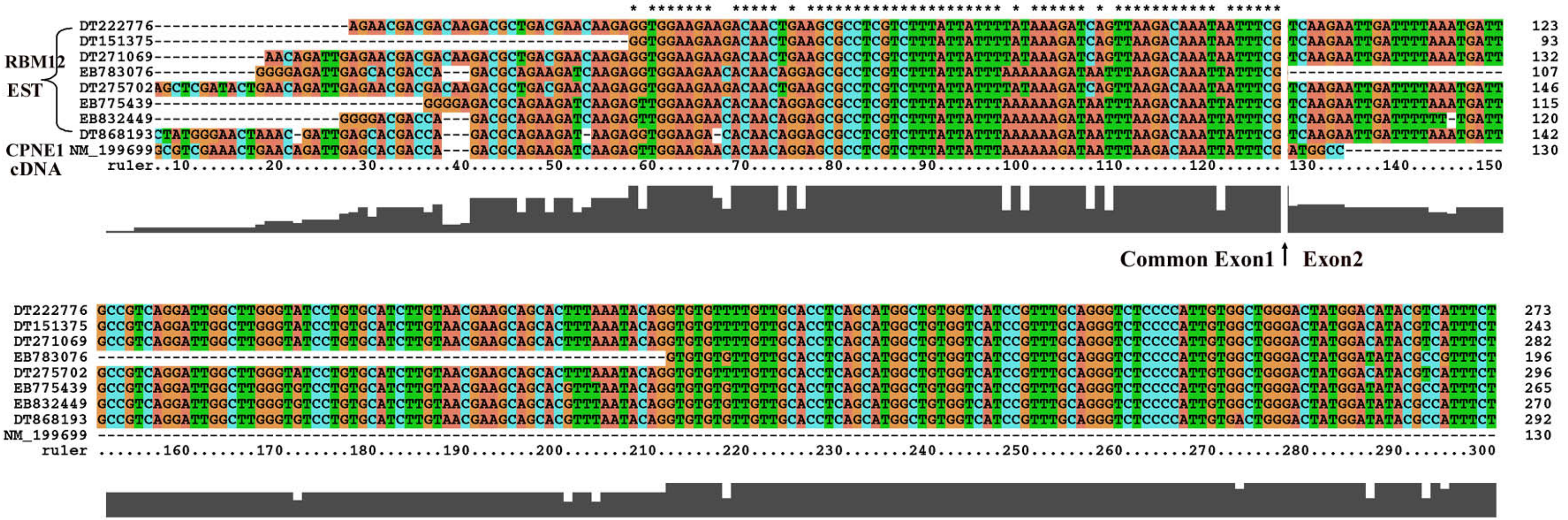

Supple. Fig3A. alignment of Human RBM12 and RBM12B: Better conservation on the N- and C- terminus

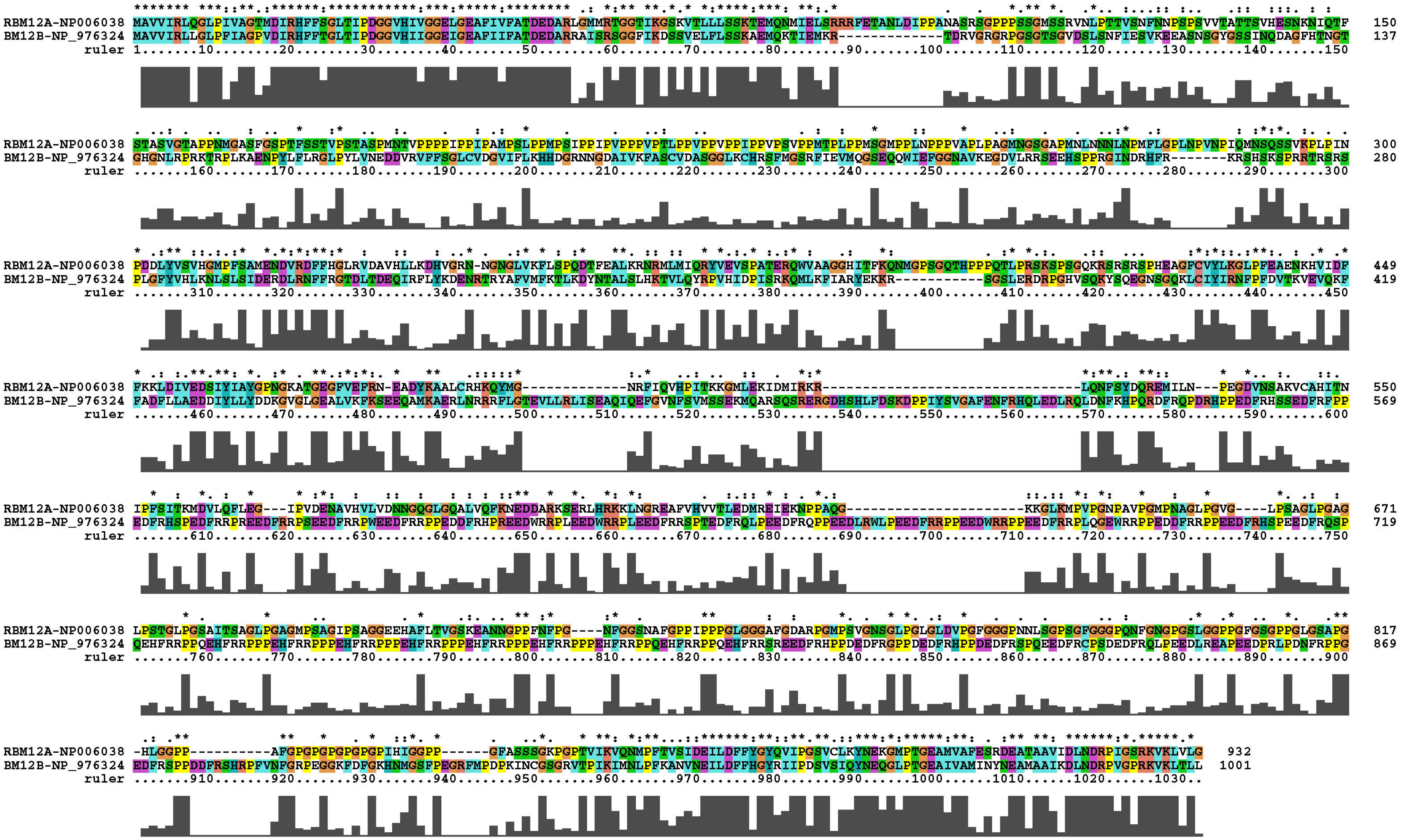

Supplementary Figure 3B. Alignment of RBM12 Orthologs and high conservation on the N-terminus

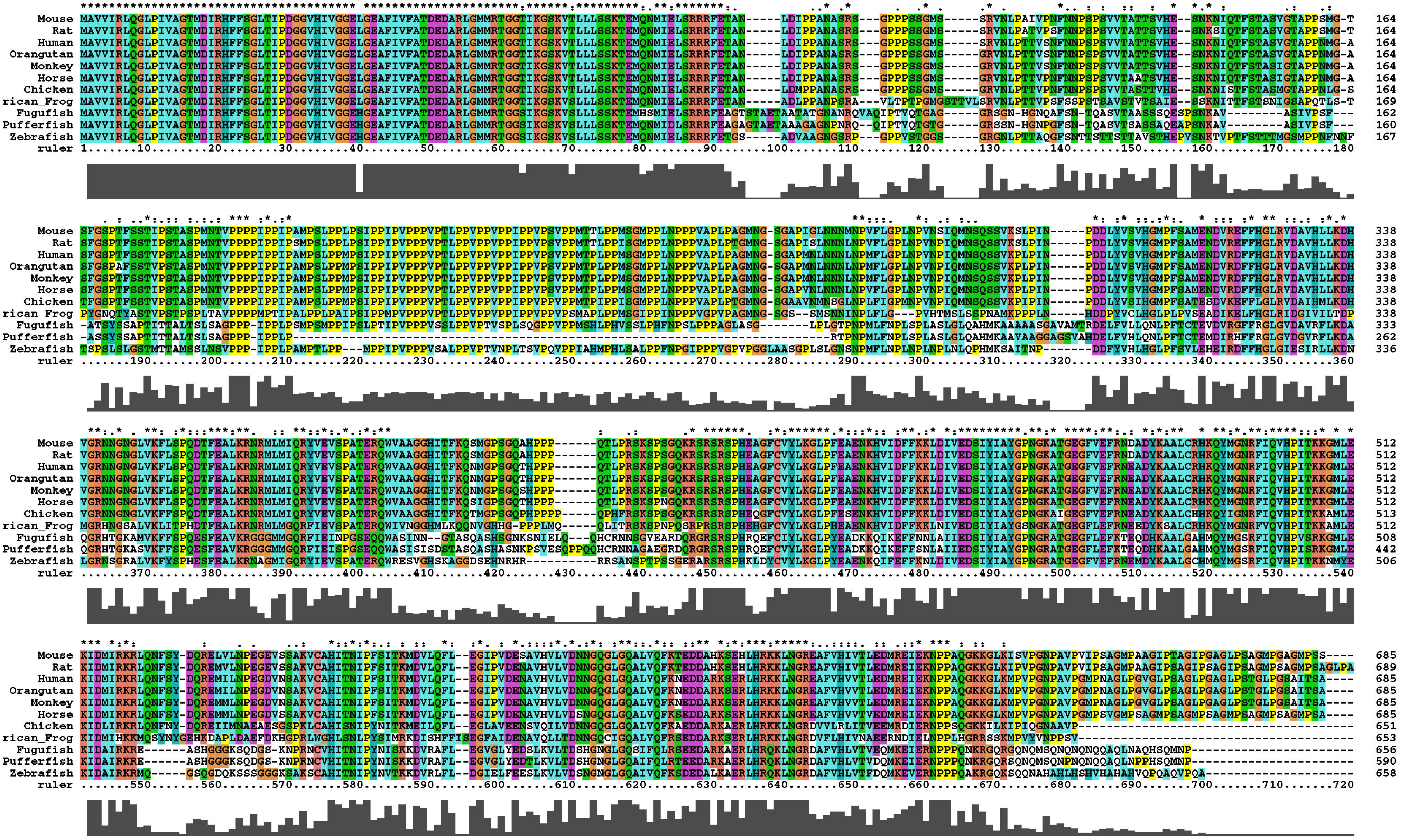

Supple. Fig 3B-continued: Better conservation on the c-terminus for RBM12 Orthologs

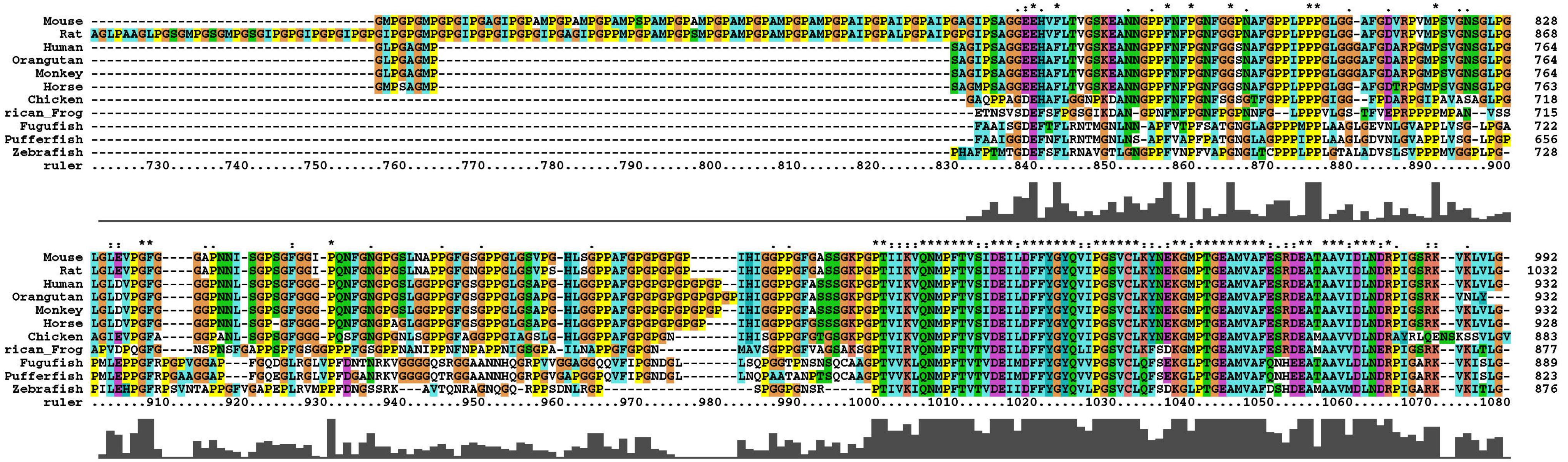

Supple. Fig4: Stem-loop formation of mammal 5'UTR sequences for the two genes.

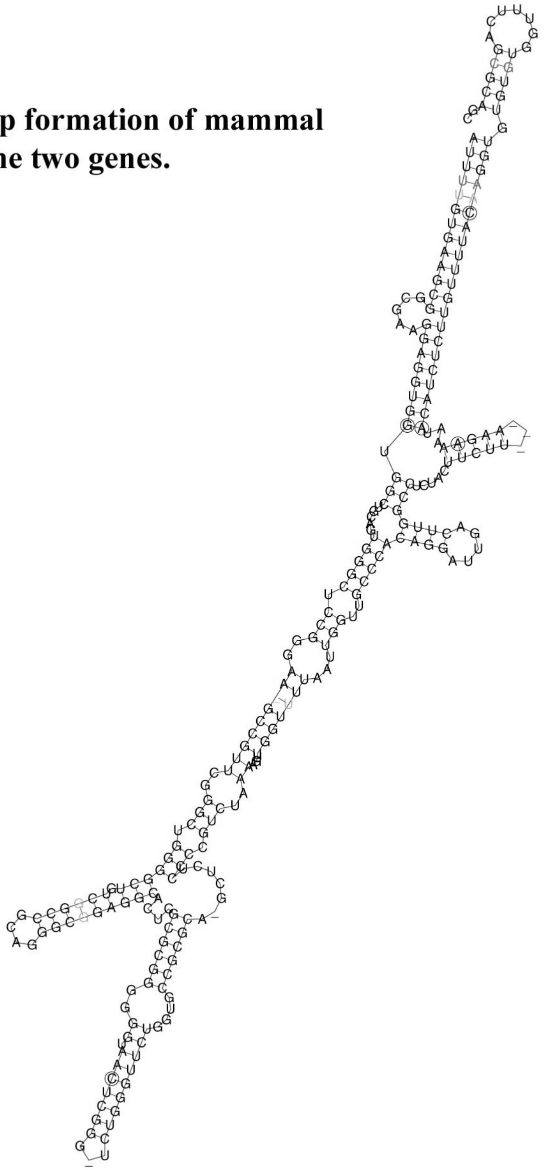

5'UTR sequences from several mammals were aligned using ClustalX and a consensus secondary RNA structure formation was predicted using Alifold program. The sequences used include sequences from exon 1, exon2 and non-coding part of exon3, which represent the longest 5'UTR sequences in RBM12 gene.

Supplementary Figure 5.

Sequence alignment for the region upstream of CPNE1/RBM12 gene pair cDNA (1000 bp) from human, mouse, rat and chicken using MAVID program (http://baboon.math.berkeley.edu/mavid/)

Bray N and Pachter L, MAVID: Constrained ancestral alignment of multiple sequences,, *Genome Research*, 14:693-699 (2004).

```
Mouse   CTTCC-----------TTCCTTTCTTCCT-----------TTCTCTCTCTCTCTCT---C
Human   TTTCTGAGACGAAGTTTCGCTTTTGTCATCCAGGGTGGAGTGCAATGGCGCCATCTGGGC
Rat     CCTCCCCAATCACCCCCTTCCT---CCTGCACAGGCGGCATAGTCTG----------G--
chicken CCCCTGGGAGCTTCCATTGGCTGTGCCTGGAGTGCATGCATGTGCTGACTTTGTTGGG--
        1.......10........20........30........40........50.........

Mouse   TCTCT-----CTCTCTCTCTCTCTCTCTCTCTCTCTCTCTCTCTCTCTCTCTCT------
Human   TCACTGCAACCTCCACCTCCCGGGTTCAAGCGATTCTCCTGCCTCAGCCTCCCAAGTAGC
Rat     ----TGCAGCCT------------------------------------------------
chicken ----TGCCACTTGT--------------------------------------------GA
        61......70........80........90........100.......110........

Mouse   --------------------CTCTCTCTCTTTCTTTCTTTCTTTCTTTCTTTCTTTCTT
Human   TGGGACCACAAGCACCGCCACCACACCCAGCTAGTT------------------------
Rat     -AGGACTTCAGGTATAATCAGATACCAGGGATTAT---------------ATGGGCGGCCA
chicken TAAGAACAAATGAATGACAAGATACCAGGG-TGCT---------------ACAGGGTGCAA
        121.....130.......140.......150.......160.......170........

Mouse   TCTTTCTTTCTTTCTTTCTGTGTGTGTTTAAGACAGGGTTTCTCTGTGTAGTGCTGGCTG
Human   --------------TTTGTATTTTTAGTAGAGGCTGGGTTTTGCCTTGTTGGCCAGACCG
Rat     GGAGAGATTAAAAGACATTTTCCGGGCTGGAGAGATGGCTCAGCGGTTAAGAGCA-----
chicken ACAGGGA---------GCTGCGTGTGGTGCACAGAGGGCCCAAC---TTGGCCCT-----
        181.....190.......200.......210.......220.......230........

Mouse   GTCTCAAACTCAGAA-------ATCTGCCTGCCTCTGCCTCCCAA-GTGCGGGGATTAGA
Human   GTCTCGAACTCCTGACCTCATGATCCGCCCTTCTCGGACTCCCAAAGTGCTGGGATTATA
Rat     ---------------------------CCCGACTACTCTTCCAGAGGTCCTGAGTTCAGT
chicken ---------------------------CTTGGCTAAGGAGACAAAAGGGAGGGGTTCACT
        241.....250.......260.......270.......280.......290........

Mouse   GGCTTGTGCCACCACTGCCAAGCAGGATTATTTTCTCTTTCTTTCTTTCT----------
Human   GGCGTGAGCCACCGCGCTCGGCCTGTATAA---------ACTTTTTTTT----------
Rat     TCCC--AGCAACCACATGGTGGCTCA-CAACCATCT-------------------GTA
chicken TGAC--AGAAGGGATACATTTTCAGAGCAACCTTATCAGAAATAAAGTCTAAAAGCAGTA
        301.....310.......320.......330.......340.......350........

Mouse   ----------------------------------TTCTTTCTTTCTTTCTTTCTTTCTT
Human   ----------------------------------TTTTTTTTTTTTTTTGAGATGGAG
Rat     AAG--------------AGATCCGATGCCGGGGGTTGGGGATTTAGCTCAGTGGTAGAG
chicken AAGCCAGGGTTACCAAGGAGATGAGCAGGTGGGGGCTGTTTATT---GGAAGGGGTTGAG
        361.....370.......380.......390.......400.......410........

Mouse   -------------------------TCTTTCTTTCTTTCTTTCTTTCTTTCTTTCTTTCT
Human   -------------------------TCTTGC--TCTTTCGCCCAGGCCGGAGTGCAGTGG
Rat     CGCTTGCCTAGCAAGC---ACAAGGCCTGGGTTCGGTCCCCAGCTCCGAAAAAAAGAAA
chicken AG--TGGTGAGGAAGAGTAAGAAGGGGCTCTGTGAACATCCCAGCGCC------------
        421.....430.......440.......450.......460.......470........

Mouse   TTCTTTCTTTCTTTCTTTCTTTCTCTTCCTTCCTTCCTTCCTTTTTCTTTCTTTCTTTCT
Human   CGCAATCTCGGCTCACTGCAAGCTCCGCCTCCCAGGTTCACGCTAT--------------
Rat     AAAAAAAGAGATTCGATGCC--CTCTTC--------------------------------
chicken ----------------TGCCAGCACTGC--------------------------------
        481.....490.......500.......510.......520.......530........

Mouse   TTCTTTTCTTTCTCTCTCTTTCTTTCTCTTTCTTTCTTTCT-----------TTCTTTCT
Human   --------------TCTCCTGCCTCAGCCTCCCGAGTAGCTGGGACTACAGGTGCCCGCC
Rat     ---------------------------------TGGTGTATCTGAAGACAGCTACAGTGT
chicken ---------------------------------TGGTTGGTGCTGAG-CAGCAGCCGAGC
        541.....550.......560.......570.......580.......590........

Mouse   TTCTTTCTTTCTTTCTTTCTTTCTTTCTTTCTTTCTTTCTTTCTTTCTTTCTTTCTTTCT
Human   ACCACGC-----------------------------------------------------
Rat     ACTTATAT---------------ATAAAAAATGAATAAAT---CTTTAAAAAAAGACATT
chicken TGTCACAATCCTGGCTTTGCTGGATAAAAA-------------GTTTGCTGTATGACTGT
        601.....610.......620.......630.......640.......650........

Mouse   TTCTTTCTTTCTTTCTTTCTTTCTTTCTTTCTTTCTTTCTTTCTTTCTTTCTTTCTTTTT
Human   ------------------------------------CCGGCTAATTTTTTGTATTTTT
Rat     TTCCTTCTTTTTATA---TTTGTTCTTTATATTTATATCTATTTTCAATTTTAATTTTATT
chicken GTGCTCCGTGCAGCAGACTGTGCTGGTTGTGTTT---TCTATTGGAAGTGAGA-------
        661.....670.......680.......690.......700.......710........
```

```
Mouse    TGGTTTTTCGAGACAGGGTTTCTCTGTATAGTCCTGGCTGTCCTG--------------G
Human    TAG----AAGAGACGGGGTTTCACCGTGTTAGCCAGGATGGTCTC--------------G
Rat      AATGTCTCTGTGTAAGAGTTTGTGCACATGAGTGAAGATGTCTTCAGAGGCCAGAGGAGG
chicken  --------GCTGGCACAGTCTGTTCCATGGAGGCACAGTGCTGGCGGAGGTGCCTCGCTG
         721.....730.......740.......750.......760.......770........

Mouse    AACTCACTCTGTAGCCCAGGCTGGCCTCGAACTCAGAATTCGCCTGCCTCTGCCTCCCA-
Human    ATCT--------------------CCCGACCTCGTGATCCGCCCGCCTCGGCCTCCCAA
Rat      GCGTCAGATTTGAGGCCCGAGTTACTTGAGGTT--------------GTGAGCAGCTCAA
chicken  TGCTCCCAT-------CAGGGTGAGCTCTGGTT--------------GTGTGCA------
         781.....790.......800.......810.......820.......830........

Mouse    --------------------------------------------T----------TTTT
Human    AGTGCTGGGATTACAGGCGTGAGCCACCGCGCCCGGCCCCCGGCCT----------GTAT
Rat      GACTTCGTGAAGAGCAGTATGAGCTTTTATCCTTTGAGCCTTTTCTCCACCCCAAGGCAA
chicken  --------------CAGCAAGAACGTTTAAC---TGAG------------CGAAGGGTG
         841.....850.......860.......870.......880.......890........

Mouse    TTTTTTTTCAATAGTCAAAAAAAGGTTCAAGCTTTTGCTTCTT----------------
Human    AAACTCTTAAATGG--GAAAAAAGAATCAGGTTTTGGCTGTTTTGTCAGGATATAATAAC
Rat      AATATTTTCAATAGCCAAAAAAAAACAAAA---------------------CAAAACAG
chicken  AAT-------GCAGCAAAGGGGACTTACAA---------------------CAAAAAGG
         901.....910.......920.......930.......940.......950........

Mouse    ---------------GGTGCG-------TTTAATCCTCAAGACAGTTCTGCAGTGAAGGC
Human    TAGCCACCACGTTGTGGTCCA-------TTTAATCCTCAAAAAACTT------AGAGGTA
Rat      GACCTTTTGCTTGGTGGTGCG-------TTTAATCCTGAAGACAGTTCTGCAGTGTAGGC
chicken  GCACTGACTCGGGGTAGTGTGGCACAGCTCTAATTCT---GCCACCTTCTAAGTGAAGAA
         961.....970.......980.......990.......1000......1010.......

Mouse    ATTGTTTTTCTC-CCTTCACGGGGGTCTCCAAG---------------AGCCAAAGGAG
Human    AGCACAATTCGCTCCTTCCGCGGAAACTGGAAG---------------ACGTGAAGCA-
Rat      ACAC-----------TTTCACGGGGGCTCTAA---------------AAGCCAAAGGAA
chicken  GCAGCA----GCTTGTTGCCTCAGGGCTCTACGTTCGTTGCCAATCTGATGCCTCTGGTT
         1021.....1030......1040......1050......1060......1070.......

Mouse    CCTGTACTTTAAAAGCTCTGCAAGGAA---------------------------------
Human    -----ATTTGCACAGTCTTGCAGTGAA---------------GGGCCAAGCTAGACACT
Rat      CCTGTAC---AAAAGCTCTGCAGCGAACCT-----------------------------
chicken  ACTCAGC---AGTAACCCTTCCGCTCACTTGTACCACCGTTAATTTCCAGAAAACTCAAC
         1081.....1090......1100......1110......1120......1130.......

Mouse    ----------------CCTTGGACAATAGC-------------------TTCAGGTCT
Human    CATACCCAGCCCACCCGCCAGGGCCACTAGC-------------------CCAAGGTCG
Rat      -------------------TGGACAATAGC-------------------TTCAGGCCG
chicken  ATATCATCGATGTATACTATTGTACGAGAGGCAACGATACGTATCGTGCGATACAGGTCT
         1141.....1150......1160......1170......1180......1190.......

Mouse    GACTTT-------------GCAAACAGCTACTAAA-ATACCGGGTTCACCA-----CAAA
Human    GTCCTGTGCGCCAACAGTCGCAAGCAGGGACTGAATATCCCCCTCTTAGCA-----CAAA
Rat      GACTCTGCA-------------AACAGCAATTAAAAAGCCGGGTTTA-------GCCAAA
chicken  GACGGTGCTCTGTTCAGCCCCTAACTGGACATCAAACCCCGCTGTGGTACCGGCGCGGCA
         1201.....1210......1220......1230......1240......1250.......

Mouse    TACCTGCAACTTCCGAGCTTTTGCCCCCACCCCA-----AATCCTCAGCGCGCCTGCGTC
Human    ACCCAGTAACTTCCGGGAGAT------CGCCCGA-----GACCGTCAGCGCGCTTGCGCG
Rat      TACCTGCAACTTCCAAAGTTTCCCCCT--CCCCACTCCGAAACCTCAGCGCGCCTGCGTC
chicken  CAGCCCACCTCACACACACACCCCCTGACGCGAGGCGCGAACCGCGCTGCGCATGCGCC
         1261.....1270......1280......1290......1300......1310.......

Mouse    TCCCATCAG--------------------------GTGACGTCGGCAGCGGCGCCCCGC
Human    GCCCCT-----------------------------TGACGTCAACCACGCCGTTCCTC
Rat      TCCCACCAG--------------------------GTGACGTCGGTAGCGGCGCTCCGC
chicken  ATCCACCACTGCGCGCAGCCCCACCCCGGGCAGGGAGAGACGGCCGTAGGGGGGCGCTGC
         1321.....1330......1340......1350......1360......1370.......

Mouse    C-----------------------------------------------------------
Human    CCCCTTCTGGAA------------------------------------------------
Rat      ---------------------------------------------------CCACTCTGG
chicken  GCGCGCGGCGGAGCCCCGCCCTCCTCCTTCCGCCGGGCCCTCAGCCCCGCCCCTCCCTGG
         1381.....1390......1400......1410......1420......1430.......
```

```
Mouse   ------------------------------T
Human   -----------------------------CG
Rat     GAACGCGGAGCGG-------GCG-GGCGGCC
chicken CGGCGGGTGGCGGCGCCGCAGCGAGGCGGCC
        1441.....1450......1460........
```

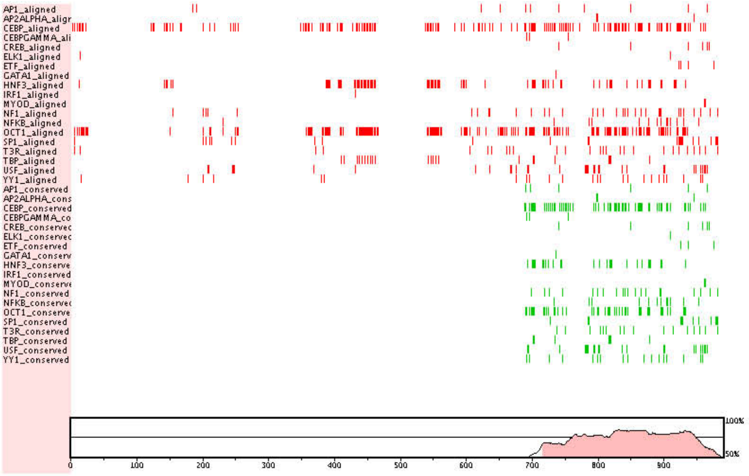

A. Mouse-rat pair

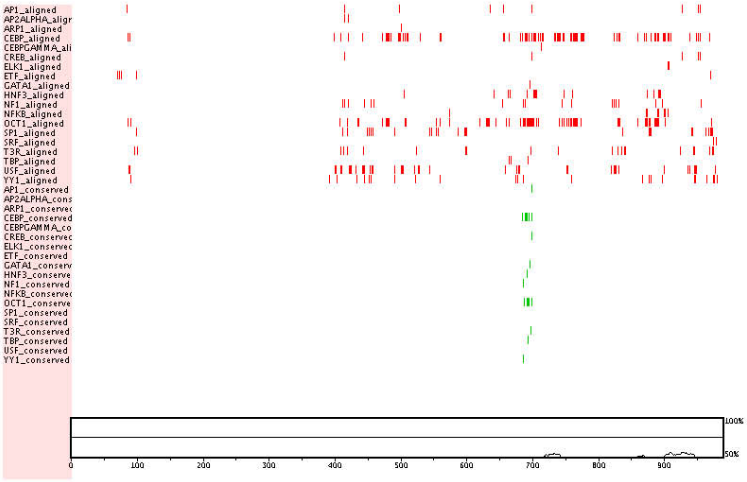

C. Human-rat pair

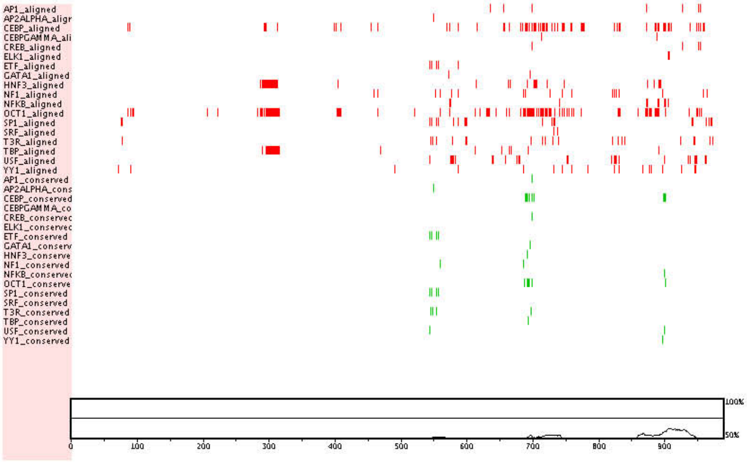

B. Human-mouse pair

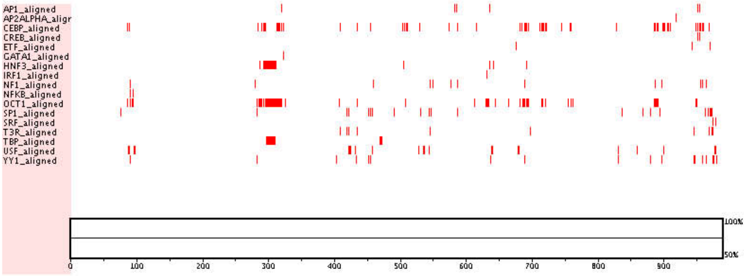

D. Human-chicken pair

Supplementary Figure 6.

Transcription factor binding sites detection in the promoter regions of CPNE1/RBM12 in human, mouse, rat, and chicken.

1,000 bp DNA sequences upstream CPNE1/RBM12 cDNA were used in transcription factor binding sites analysis using rVISTA (http://genome.lbl.gov/vista/index.shtml). rVISTA combines searching the major transcription binding site database TRANSFAC from Biobase with a comparative sequence analysis. The program first identifies potential transcription factor binding sites in each of the two aligned sequences, and determines which of the predicted sites are aligned and conserved between the species in the alignment.

X-axis is the nucleotide sequence in the first sequence, with 1000 the nucleotide immediately 5' to the first nucleotide of CPNE1/RBM12 cDNA.
In each figure, the lower panel is the sequence alignment index between the sequence pairs, while the upper panel is the identified transcription factor binding sites, with the red color for aligned TF binding sites and the green color conserved TF binding sites.

Aligned binding sites: all the binding sites in the two sequences were identified independently, but only those with core binding site positions corresponded in the alignment of the two sequences were selected as aligned binding sites.

Conserved binding sites: predicted binding sites located in the sequence fragments conserved between two species at the level of over 80% over 24 bp window were selected as conserved binding sites.

Supplementary Fig 7. cDNA sequences that align with human RBM12 cDNA, NM_006047 These cDNA sequences all have very close 5' transcription initiation sites, usually within 40bp of each other. This indicates that the sharing of most 5' exon probably reflects the sharing of the same promoter. (The ones with longer 5' sequences on the display are mostly aligned genomic sequences).

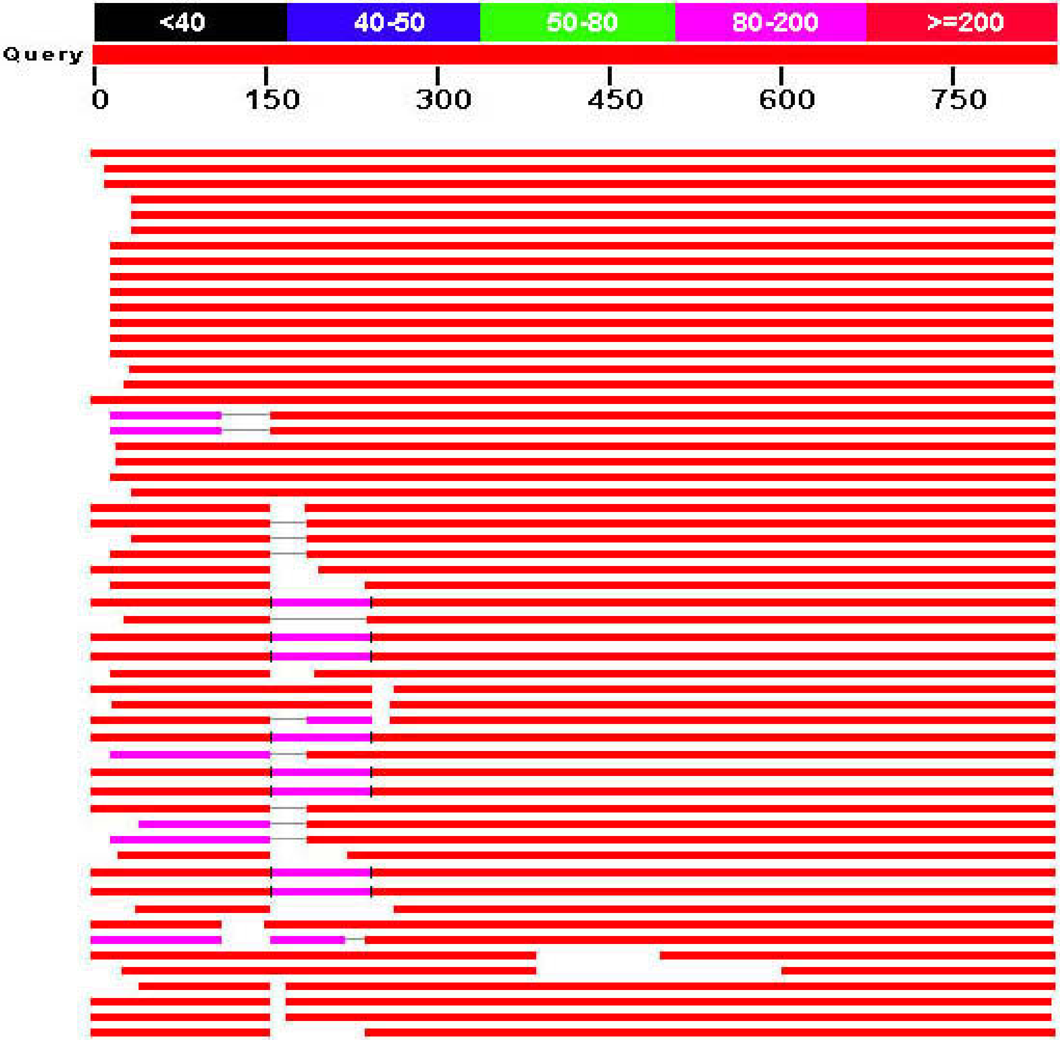

Supplement: Additional file 1 — Additional Figures 1–7. [file 1471-2164-9-456-S1.pdf]
